# Induction of transferrin receptor 1-mediated ferroptosis by ultrasound-triggered microbubble destruction sensitizes glioblastoma to radiotherapy

**DOI:** 10.1186/s43556-026-00572-8

**Published:** 2026-09-18

**Authors:** Ying He, Xunhu Dong, Xiaolu Lu, Qiong Zhu, Qingqing Miao, Zexuan Yan, Qingning Zhao, Hongying Chen, Min Qin, Yuehua Yi, Jiaqi Li, Dan Yang, Hanyun Zhang, Yali Xu, Mingliang Chen, Xiuwu Bian, Xing Hua

**Affiliations:** 1 Institute of Pathology and Southwest Cancer Centre, Southwest Hospital, Army Medical University (Third Military Medical University) and the Key Laboratory of Tumor Immunopathology, The Ministry of Education of China, Chongqing, 400038 China; 2 Department of Ultrasound, Xinqiao Hospital, Army Medical University (Third Military Medical University), Chongqing, 400037 China; 3 Department of Clinical Nutrition, Southwest Hospital, Army Medical University (Third Military Medical University), Chongqing, 400038 China; 4 Institute of Toxicology, School of Military Preventive Medicine, Army Medical University (Third Military Medical University), Chongqing, 400038 China; 5 Institute of Burn Research, State Key Laboratory of Trauma, Burn and Combined Injury, Southwest Hospital, Third Military Medical University (Army Medical University), Chongqing, 400038 China; 6 School of Basic Medical Sciences, Southern Medical University, Guangzhou, 510151 China; 7 Department of Ultrasound of Jiangbei Campus, Southwest Hospital, Army Medical University (Third Military Medical University), Chongqing, 400020 China; 8 School of Basic Medical Sciences, Army Medical University (Third Military Medical University), Chongqing, 400038 China

**Keywords:** UTMD, Radiosensitization, TFR1, Ferroptosis, Glioblastoma

## Abstract

Ultrasound-triggered microbubble destruction (UTMD), a non-invasive technique, has been proposed as a promising means of improving the efficacy of radiotherapy against glioblastoma (GBM); however, the underlying mechanisms remain to be fully clarified. Here, UTMD was shown to induce ferroptosis, an iron-dependent form of regulated cell death, in ionizing radiation (IR)-treated GL261 and 1016B cells, as evidenced by decreased cell viability, glutathione, and glutathione peroxidase 4 levels, and increased total reactive oxygen species (ROS), lipid ROS, iron/Fe^2+^, malondialdehyde contents, acyl-CoA synthetase long-chain family member 4 expression and cell death. Suppression of ferroptosis by ferrostatin-1 or deferoxamine abolished UTMD-induced radiosensitization in GBM cells, whereas blockade of apoptosis, autophagy, or necroptosis showed no significant effect. Conversely, induction of ferroptosis using erastin, RAS-selective lethal 3, and ferric ammonium citrate further promoted UTMD-mediated radiosensitivity of GBM cells. Mechanistically, UTMD markedly increased transferrin receptor 1 (TFR1) expression in IR-exposed GBM cells. TFR1 downregulation reversed UTMD-caused increase of intracellular Fe^2+^, subsequently inhibiting UTMD-induced ferroptosis and radiosensitivity of GBM cells, which were augmented by TFR1 overexpression. In GBM-bearing C57BL/6J and NOD-SCID mouse models, UTMD similarly upregulated TFR1 expression, triggered ferroptosis, and sensitized tumors to IR, and these effects were reversed by TFR1 knockdown. In conclusion, UTMD promoted the radiosensitivity of GBM partially by induction of TFR1-mediated ferroptosis, providing novel insights into the mechanism underlying UTMD-mediated radiosensitization.

**Supplementary Information:**

The online version contains supplementary material available at https://doi.org/10.1186/s43556-026-00572-8.

## Introduction

Glioblastoma multiforme (GBM, WHO grade IV), the most aggressive and malignant glioma, is associated with a dismal prognosis. Following maximal surgical resection, ionizing radiation (IR) remains a cornerstone of GBM treatment. However, therapeutic efficacy is often compromised by the intrinsic radioresistance of glioma cells, contributing to a median survival of merely 15 months [1]. Consequently, the development of safe and potent radiosensitizers is of paramount importance to improve patient outcomes. As a noninvasive therapeutic modality, ultrasound-triggered microbubble destruction (UTMD) uses acoustic cavitation of microbubbles (MBs) to locally perturb cell membrane integrity and enhance capillary permeability, and has therefore been proposed as a candidate radiosensitizer for GBM [2–4]. However, the underlying mechanisms remain to be elucidated.

Ferroptosis is a unique, iron-regulated programmed cell death pathway characterized by disturbances in glutathione (GSH) metabolism, iron overload, and redox imbalance. It exhibits distinct genetic, biochemical, and morphological hallmarks that differentiate it from apoptosis, pyroptosis, and necroptosis [5, 6]. Accumulating evidence indicates that ferroptosis significantly modulates the therapeutic responses in numerous cancers including glioma [7]. Induction of ferroptosis by genetic or pharmacological methods has been shown to effectively inhibit GBM growth and enhance radiosensitivity [8–11]. Moreover, Li et al. identified that ultrasound (US)-induced ferroptosis facilitates the phenotypic conversion of immunologically cold tumors into hot tumors, thereby augmenting antitumor immunity in triple-negative breast cancer [12]. Sun et al. found that US significantly augmented the cytotoxic effects of oridonin against pancreatic cancer cells through ferroptosis induction [13]. These observations suggest that ferroptosis is an essential mechanism underlying the anti-cancer effect of UTMD. However, limited information is available concerning the involvement of ferroptosis in UTMD-mediated GBM radiosensitization.

Transferrin receptor 1 (TFR1), a cell-surface glycoprotein, facilitates cellular iron uptake by binding to iron-loaded transferrin [14]. TFR1 has been identified as a specific marker and key regulator of ferroptosis by governing cellular iron homeostasis. It has been found that TFR1 overexpression promotes iron influx, oxidative stress, and lipid peroxidation to induce ferroptosis, while its inhibition diminishes intracellular iron accumulation and blocks erastin-triggered ferroptosis [15, 16]. A burgeoning body of evidence suggests that TFR1 is highly expressed in diverse tumors including GBM and represents a potential therapeutic target [14, 17, 18]. Recent studies have further demonstrated that TFR1-mediated ferroptosis is critically involved in GBM growth and treatment, as brucine induces glioma cell ferroptosis by increasing intracellular iron in a TFR1-dependent manner [19]. Importantly, prior work has shown that UTMD exerts its biological effects primarily by targeting cell-surface ion channels/receptors, through which it enables precise modulation of molecular signals by controlling MB-mediated mechanical forces on cell membranes [20]. However, the impact of UTMD on TFR1 (a cell-surface receptor) remains unclear.

Accordingly, in this study, the role of TFR1-mediated ferroptosis in UTMD-induced radiosensitivity of GBM was intensively explored in vitro using cultured murine GL261 and human 1016B GBM cells and in vivo using two orthotopic GBM mouse models (C57BL/6J and NOD-SCID mice). For the first time, we found that UTMD potentiated radiotherapeutic efficacy in GBM partially by upregulating TFR1 expression, thereby increasing intracellular iron/Fe^2+^, promoting lipid peroxidation, and ultimately triggering ferroptosis. These findings provide novel mechanistic insights into UTMD-induced radiosensitization and offer new evidence for the clinical translation of UTMD as an adjuvant therapy for GBM radiotherapy.

## Results

### UTMD induced ferroptosis in IR-treated GBM cells

**Fig. 1:**
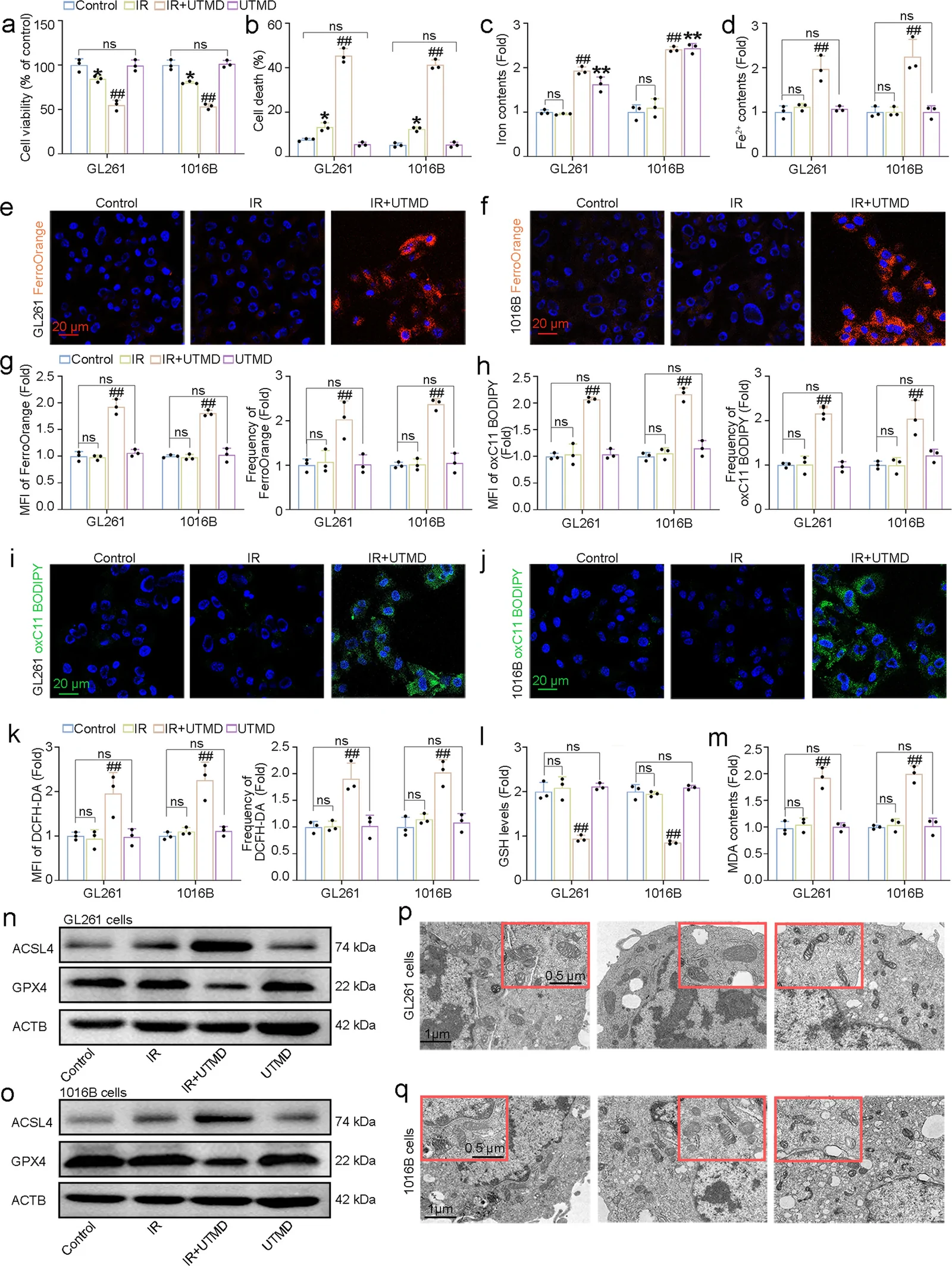
UTMD induced ferroptosis in IR-treated GBM cells. GL261 and 1016B cells were subjected to UTMD (1.2 W/cm^2^ ultrasonic intensity plus 200 μL/mL MBs, 10% duty cycle, 60 s) with or without subsequent IR (2 Gy). After 24 h, **a** Cell viability was assessed with a cell counting kit-8 (CCK-8) assay. **b** Cell death was evaluated by PI staining and flow cytometry. **c** Total iron and **d** Fe^2+^ contents were measured via a specific iron assay kit. Representative fluorescence images of intracellular Fe^2+^ levels with FerroOrange staining in **e** GL261 and **f** 1016B cells. **g** Flow cytometry analyses of the mean fluorescence intensity (MFI) and frequency of intracellular Fe^2+^ levels with FerroOrange staining. **h** Flow cytometry analyses of the MFI and frequency of lipid ROS contents (ox C11-BODIPY) with C11-BODIPY staining. Representative fluorescence images of **i** GL261 and **j** 1016B cells stained with C11-BODIPY. **k** Flow cytometry analyses of the MFI and frequency of ROS contents with DCFH-DA staining. Measurement of **l** GSH and **m** MDA levels by specific assay kits. Western blot analysis of GPX4 and ACSL4 expression in **n** GL261 and **o** 1016B cells. Mitochondrial morphological changes in **p** GL261 and **q** 1016B cells were detected by transmission electron microscopy. Data are expressed as the mean ± SD (n = 3). ^*^p < 0.05, ^**^p < 0.01 vs. the vehicle-treated control group; ^##^*p* < 0.01 vs. the IR-treated group; ns, not significant

To determine whether ferroptosis contributes to UTMD-induced radiosensitization, a comprehensive panel of established ferroptosis indicators was examined in two independent GBM cell models, murine GL261 and human 1016B cells, exposed to IR alone or to IR combined with UTMD. In line with our earlier report [3], UTMD (1.2 W/cm2 ultrasonic intensity plus 200 μL/mL MBs) significantly reduced cell viability and increased cell death (staining by propidium iodide (PI)) in IR (2 Gy)-treated GL261 and 1016B cells (Fig. 1a, b and Fig. S1a, b), confirming the radiosensitizing effect of UTMD in both cell models. We next asked whether this sensitization was accompanied by ferroptotic alterations. Compared with IR alone, the addition of UTMD markedly increased intracellular total iron contents (Fig. 1c) and Fe^2+^ levels, as evidenced by quantitative assays as well as FerroOrange fluorescence (a special intracellular Fe^2+^ probe) staining and flow cytometry analyses (Fig. 1d–g and Fig. S1c, d). UTMD also notably promoted lipid peroxidation, as reflected by increased oxidized BODIPY™ 581/591 C11 (oxC11-BODIPY, a special intracellular lipid ROS probe) signals and enhanced total reactive oxygen species (ROS) generation in IR-treated GBM cells (Fig. 1h–k and Fig. S1c, d). However, IR alone elicited only marginal changes in these parameters (Fig. 1c–k and Fig. S1c, d). In agreement with enhanced lipid peroxidation, combined UTMD and IR treatment further elevated malondialdehyde (MDA) levels and concomitantly reduced intracellular glutathione (GSH) contents relative to the single IR-treated group (Fig. 1l, m).

We then examined acyl-CoA synthetase long-chain family member 4 (ACSL4), which esterifies polyunsaturated fatty acids for incorporation into membrane phospholipids and thereby fuels lipid peroxidation, and glutathione peroxidase 4 (GPX4), which uses GSH to neutralize lipid hydroperoxides and thereby suppresses ferroptosis; both are commonly used ferroptosis markers [21]. Western blot analysis showed that UTMD notably increased ACSL4 expression and decreased GPX4 expression in IR-treated GBM cells (Fig. 1n, o). Notably, the direction and magnitude of all these changes were highly consistent between GL261 and 1016B cells, indicating that UTMD-induced ferroptosis was a shared response of GBM cells rather than a cell line-specific phenomenon.

To further corroborate UTMD-triggered ferroptosis, mitochondrial morphology (another canonical ferroptotic hallmark [21]) was scrutinized by transmission electron microscopy. As illustrated in Fig. 1p, q, UTMD evoked mitochondrial atrophy, increased membrane density, cristae depletion and outer-membrane rupture in IR-treated GBM cells. Additionally, compared with the untreated control, UTMD alone notably increased intracellular total iron contents; however, it did not significantly affect cell viability, cell death, or GPX4 and ACSL4 expression, nor did it significantly affect intracellular Fe^2+^, MDA, or GSH levels (Fig. 1a–d, g, h, k–o and Fig. S1a–d), suggesting that single UTMD treatment was insufficient to induce ferroptosis in GBM cells. Taken together, the concordant changes in iron homeostasis, redox balance, lipid peroxidation products, ferroptosis-related protein expression, and mitochondrial ultrastructure indicated that UTMD induced ferroptosis in IR-treated GBM cells.

### Ferroptosis mediated UTMD-induced radiosensitization in GBM cells

**Fig. 2:**
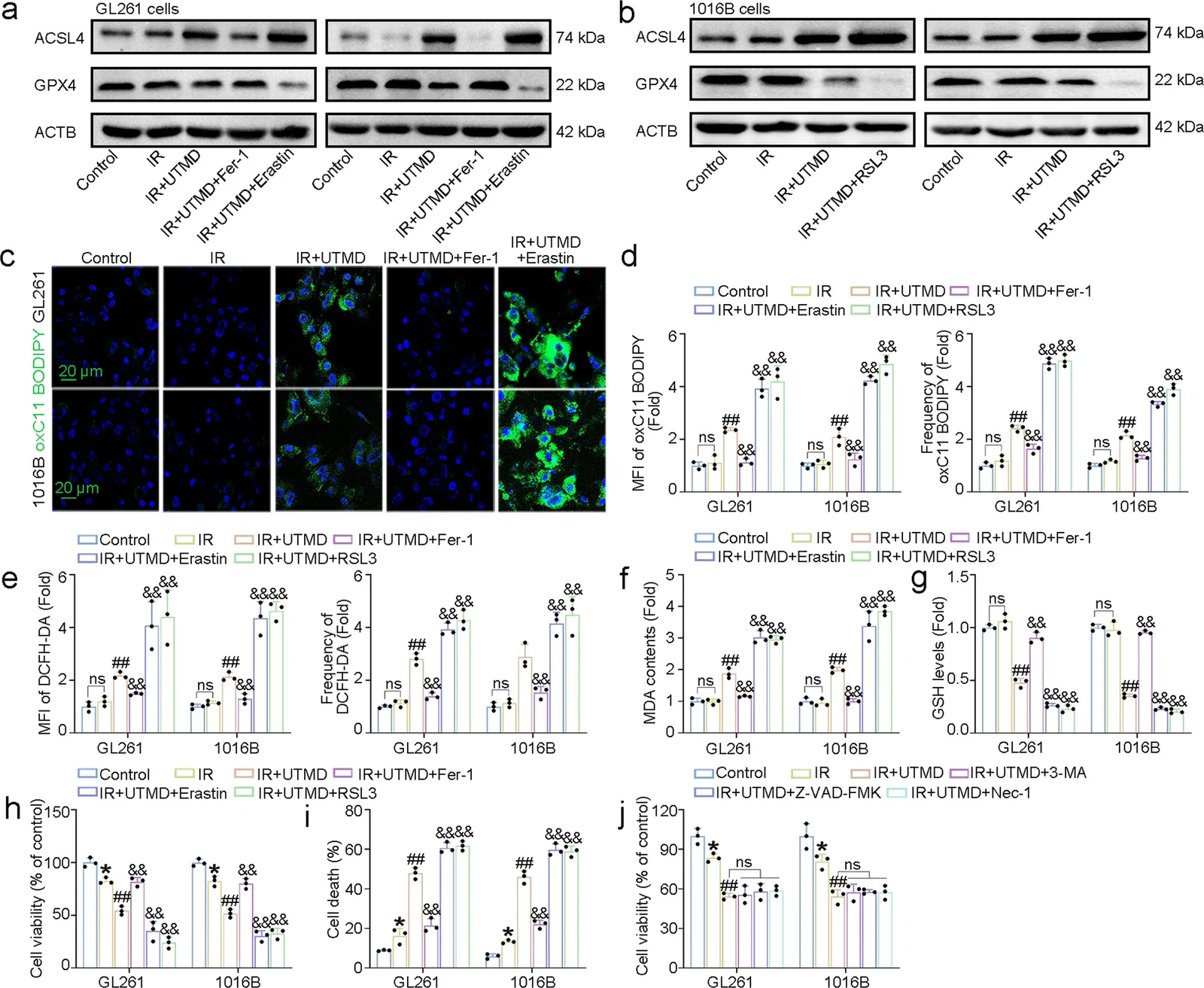
Ferroptosis mediated UTMD-induced radiosensitization in GBM cells. **a** GL261 and 1016B cells were incubated with Fer-1 (50 μM) or erastin (10 μM) for 1 h before UTMD treatment and subsequent IR (2 Gy) exposure. 24 h later, ACSL4 and GPX4 levels were detected by western blot analysis. **b** GL261 and 1016B cells were incubated with RSL3 (2.5 μM) for 1 h before UTMD treatment and subsequent IR (2 Gy) exposure. 24 h later, ACSL4 and GPX4 levels were detected by western blot analysis. **c** Representative fluorescence images of GL261 and 1016B cells stained with C11-BODIPY. **d** Flow cytometry analyses of the MFI and frequency of lipid ROS contents with C11-BODIPY staining. **e** Flow cytometry analyses of the MFI and frequency of ROS contents with DCFH-DA staining. Measurement of **f** MDA and **g** GSH levels by specific assay kits. **h** Cell viability was assessed with a CCK-8 assay. **i** Cell death was evaluated by PI staining and flow cytometry. **j** Cells were treated with Z-VAD-FMK (50 μM), 3-MA (5 mM), or Nec-1 (20 μM) for 1 h followed by IR or IR plus UTMD treatment for another 24 h. Cell viability was assessed with a CCK-8 assay. Data are expressed as the mean ± SD (n = 3); ^*^p < 0.05 vs. vehicle-treated control group; ^##^*p* < 0.01 vs. IR-treated group; ^&&^*p* < 0.01 vs. IR and UTMD co-treated group; ns, not significant

Having found that UTMD triggered ferroptosis in irradiated GBM cells, we next evaluated whether ferroptosis was causally responsible for the radiosensitizing effect of UTMD. Ferroptosis was pharmacologically suppressed or amplified with ferrostatin-1 (Fer-1, a specific ferroptosis inhibitor, 50 μM) and erastin (a specific ferroptosis inducer that depletes intracellular GSH, 10 μM), respectively. The UTMD-induced upregulation of ACSL4 and downregulation of GPX4 were significantly abolished by Fer-1 but further amplified by erastin (Fig. 2a). To rule out compound-specific artifacts, RAS-selective lethal 3 (RSL3, 2.5 μM), another potent ferroptosis inducer that directly targets GPX4, was applied as an additional positive control. As shown in Fig. 2b, RSL3 significantly enhanced UTMD-induced changes in ACSL4 and GPX4 expression in IR-treated GBM cells, phenocopying the effects of erastin. Consistent with these protein-level changes, Fer-1 markedly attenuated, whereas erastin and RSL3 further enhanced, UTMD-induced lipid ROS accumulation, total ROS generation, and MDA production as well as GSH depletion in IR-treated GBM cells (Fig. 2c-g and Fig. S1c, d). Moreover, Fer-1 notably attenuated UTMD-caused inhibition of cell viability and induction of cell death, whereas erastin and RSL3 further enhanced the radiosensitizing effect of UTMD (Fig. 2h, i and Fig. S1a, b). Comparable responses were obtained in GL261 and 1016B cells, indicating that the magnitude of ferroptosis tightly correlated with the degree of radiosensitization achieved by UTMD.

Finally, to determine whether other programmed cell death pathways were involved in UTMD-induced radiosensitization, Z-Val-Ala-Asp(OMe)-fluoromethylketone (Z-VAD-FMK, an apoptosis inhibitor, 50 μM), 3-Methyladenine (3-MA, an autophagy inhibitor, 5 mM), and necrostatin-1 (Nec-1, a necroptosis inhibitor, 20 μM) were applied. None of these inhibitors rescued the UTMD-mediated reduction of cell viability (Fig. 2j), suggesting that apoptosis, autophagy, and necroptosis were unlikely to contribute substantially to the observed radiosensitization. Collectively, the rescue by a ferroptosis inhibitor, the sensitization by two structurally unrelated ferroptosis inducers, and the lack of effect of apoptosis, autophagy, and necroptosis blockade indicated that UTMD sensitized GBM cells to radiotherapy predominantly by triggering ferroptosis.

### UTMD-induced ferroptosis depended on intracellular iron levels

**Fig. 3:**
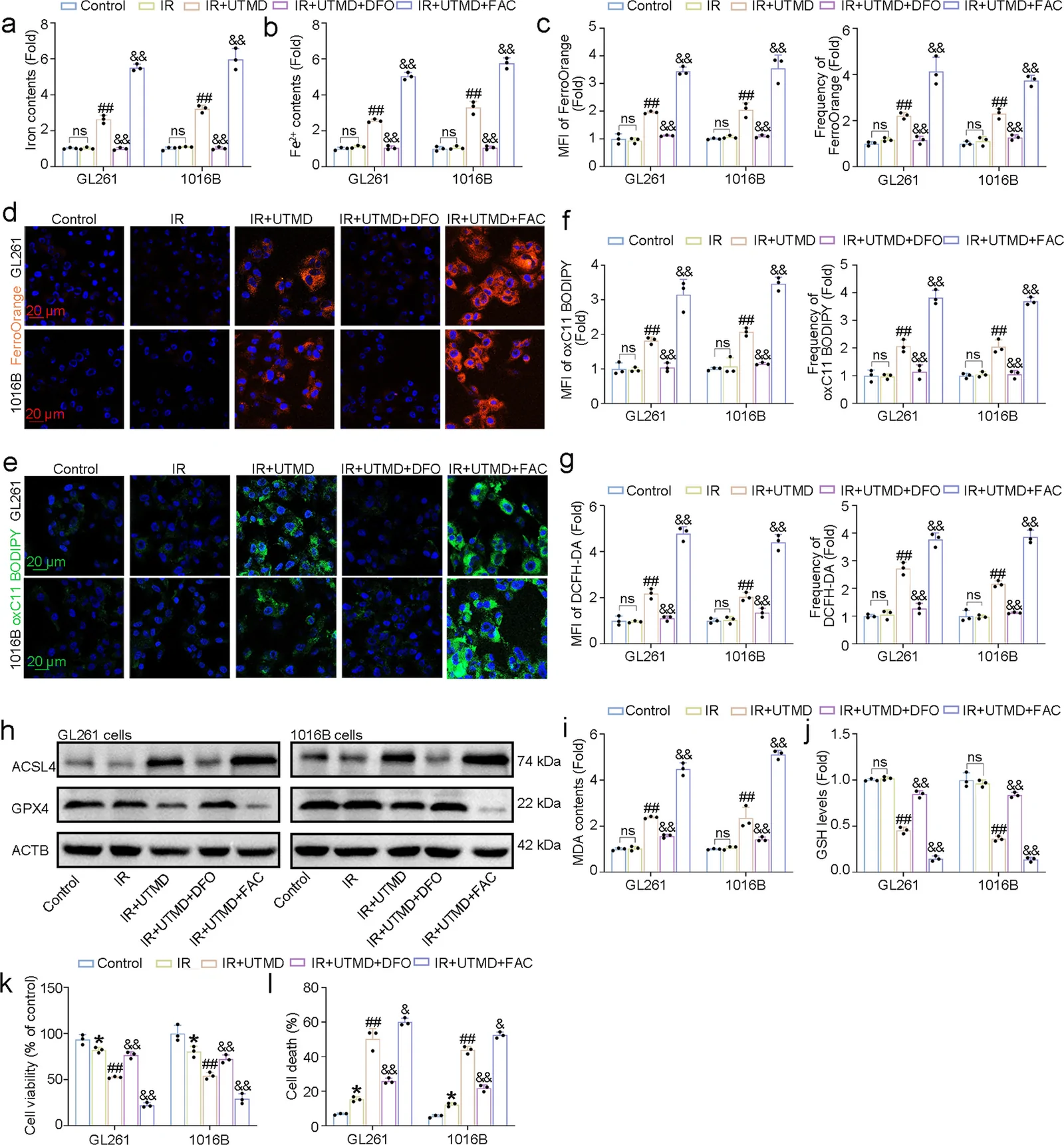
UTMD-induced ferroptosis depended on intracellular iron levels. GL261 and 1016B cells were incubated with DFO (500 μM) or FAC (500 μM) for 1 h before treatment with or without UTMD and subsequent IR (2 Gy) exposure. **a** Total iron and **b** Fe^2+^ levels were measured via a specific iron assay kit. **c** Flow cytometry analyses of the MFI and frequency of intracellular Fe^2+^ levels with FerroOrange staining. **d** Representative fluorescence images of intracellular Fe^2+^ levels with FerroOrange staining in GL261 and 1016B cells. **e** Representative fluorescence images of GL261 and 1016B cells stained with C11-BODIPY. **f** Flow cytometry analyses of the MFI and frequency of lipid ROS contents with C11-BODIPY staining. **g** Flow cytometry analyses of the MFI and frequency of ROS contents with DCFH-DA staining. **h** ACSL4 and GPX4 levels were detected by western blot analysis in GL261 and 1016B cells. Measurement of **i** MDA and **j** GSH levels by specific assay kits. **k** Cell viability was assessed with a CCK-8 assay. **l** Cell death was evaluated by PI staining and flow cytometry. Data are expressed as the mean ± SD (n = 3); ^*^p < 0.05 vs. vehicle-treated control group; ^##^*p* < 0.01 vs. IR-treated group; ^&^*p* < 0.05, ^&&^*p* < 0.01 vs. IR and UTMD co-treated group; ns, not significant

Iron overload plays a pivotal role in ferroptosis [22]. Given that UTMD increased intracellular iron contents in GBM cells, we next investigated whether iron availability determined the extent of UTMD-induced ferroptosis. Deferoxamine (DFO, a high-affinity iron chelator, 500 μM) and ferric ammonium citrate (FAC, a non-transferrin-bound iron source that promotes intracellular iron overload, 500 μM) were used to deplete or supplement intracellular iron, respectively. As shown in Fig. 3a–d and Fig. S1c, d, UTMD-caused increase of intracellular total iron and Fe^2+^ was inhibited by DFO, but was further enhanced by FAC in IR-treated GBM cells, indicating that both agents effectively modulated the intracellular iron pool under our experimental conditions.

Consistent with the notion that iron availability dictates ferroptotic intensity, DFO notably abolished UTMD-induced lipid ROS, total ROS, and MDA production, ACSL4 upregulation, GPX4 downregulation, GSH depletion, and cell death (Fig. 3e–l and Fig. S1a–d). In contrast, FAC substantially augmented UTMD-induced production of lipid ROS, total ROS, and MDA, further increased ACSL4 expression, decreased GPX4 expression and GSH contents, and enhanced UTMD-induced death of IR-treated GBM cells (Fig. 3e–l and Fig. S1a–d). These results collectively indicated that UTMD-induced ferroptosis and radiosensitization of GBM cells were positively modulated by intracellular iron contents.

### UTMD induced ferroptosis in a TFR1 dependent manner in IR-treated GBM cells

**Fig. 4:**
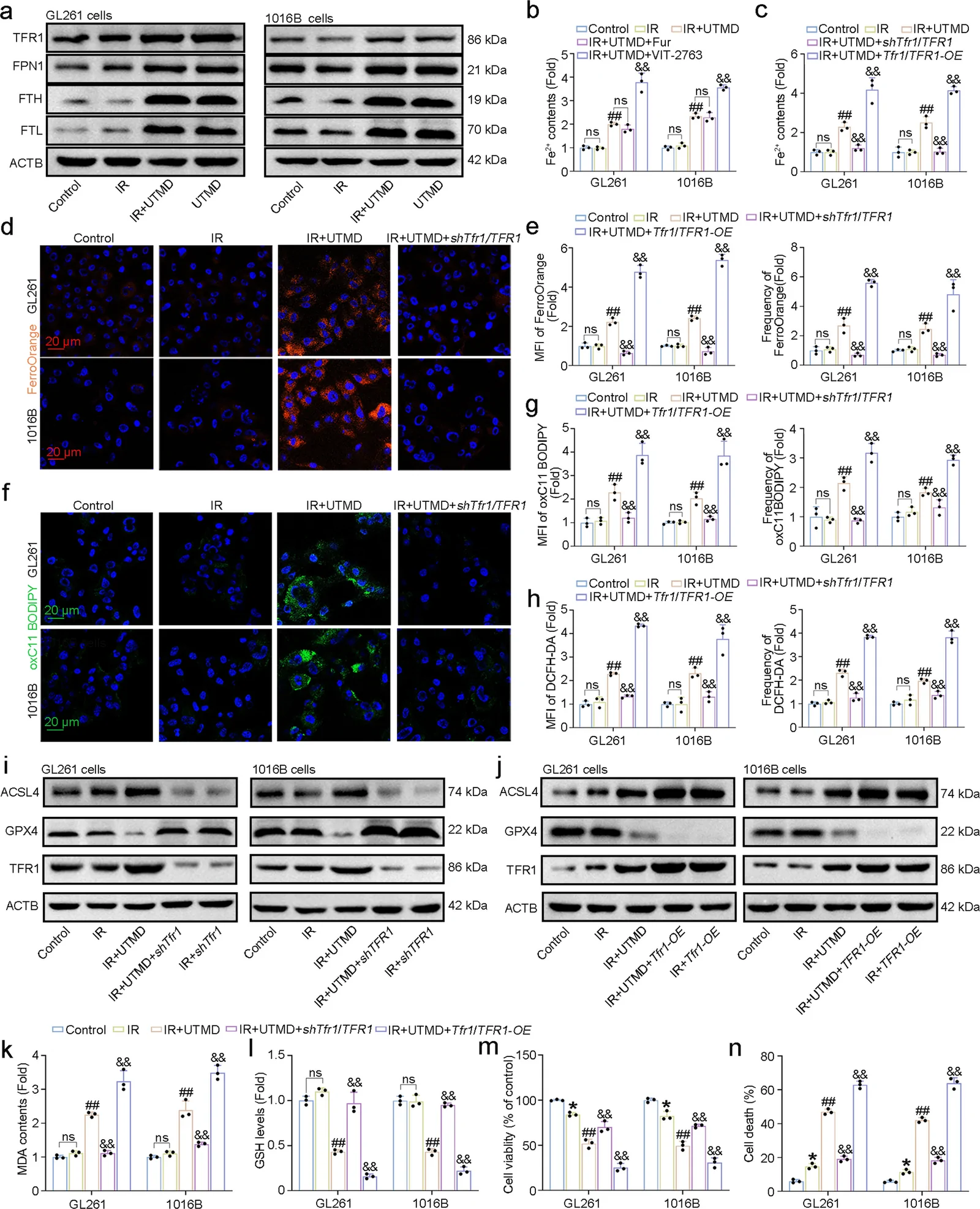
UTMD induced ferroptosis in a TFR1 dependent manner in IR-treated GBM cells. **a** GL261 and 1016B cells were treated with UTMD, and then cells were treated with or without IR (2 Gy). TFR1, FTH, FTL, and FPN1 expression was measured by western blotting. **b** GL261 and 1016B cells were incubated with VIT-2763 (2 μM) or Fur (10 μM) for 1 h before IR or IR plus UTMD treatment and were analyzed 24 h later. Fe^2+^ levels were measured via a specific iron assay kit. GL261 and 1016B cells were transfected with lentiviral vectors carrying *shTfr1/TFR1 or Tfr1/TFR1-OE* as described in the Materials and Methods. Then, the cells were treated with UTMD followed by IR (2 Gy) exposure. **c** Fe^2+^ levels were measured via a specific iron assay kit. **d** Representative fluorescence images of intracellular Fe^2+^ levels with FerroOrange staining in GL261 and 1016B cells. **e** Flow cytometry analyses of the MFI and frequency of intracellular Fe^2+^ levels with FerroOrange staining. **f** Representative fluorescence images of GL261 and 1016B cells stained with C11-BODIPY. **g** Flow cytometry analyses of the MFI and frequency of lipid ROS contents with C11-BODIPY staining. **h** Flow cytometry analyses of the MFI and frequency of ROS contents with DCFH-DA staining. **i** and **j** TFR1, ACSL4 and GPX4 expression was detected by western blot analysis in GL261 and 1016B cells. Measurement of **k** MDA and **l** GSH levels by specific assay kits. **m** Cell viability was assessed with a CCK-8 assay. **n** Cell death was evaluated by PI staining and flow cytometry. Data are expressed as the mean ± SD (n = 3); ^*^p < 0.05 vs. vehicle-treated control group; ^##^*p* < 0.01 vs. IR-treated group; ^&&^*p* < 0.01 vs. IR and UTMD co-treated group; ns, not significant

The above results prompted us to identify the route through which UTMD expands the intracellular iron pool. Cellular iron homeostasis is orchestrated by the coordinated action of iron import, storage, and export proteins, among which TFR1, a key ferroptosis regulator, mediates the cellular uptake of transferrin-bound iron and thereby enlarges the labile iron pool, consequently driving ROS production and lipid peroxidation [23]. We therefore profiled the expression of the principal iron-handling proteins in GBM cells. As illustrated in Fig. 4a, UTMD significantly increased TFR1 expression, indicating that UTMD upregulated the iron-uptake machinery irrespective of irradiation. Intriguingly, UTMD also elevated ferroportin 1 (FPN1, a vital iron export transporter that regulates cellular iron release) and ferritin (a protein complex composed of ferritin heavy chain (FTH) and ferritin light chain (FTL) that binds intracellular Fe^2+^) expression. Because ferritin sequesters excess intracellular iron and FPN1 extrudes it, the upregulation of these two proteins was most likely a compensatory, cytoprotective response to iron overload rather than the cause of iron accumulation, whereas the concurrent induction of TFR1 implicated enhanced iron uptake as the initiating event underlying iron overload in GBM cells. To functionally dissect the contribution of iron export to UTMD-induced iron overload, we employed VIT-2763 (2 μM), a specific FPN1 inhibitor that blocks cellular iron efflux, and fursultiamine (Fur, an antagonist of hepcidin, 10 μM), which stabilizes FPN1 on the cell surface by suppressing the FPN1-hepcidin interaction, thereby enhancing iron export. As shown in Fig. 4b, VIT-2763 further amplified UTMD-induced increase of intracellular Fe^2+^; however, Fur did not significantly affect this increase. These results suggested that UTMD caused intracellular iron overload by enhancing TFR1-dependent iron uptake rather than by suppressing ferritin or FPN1.

To directly define the role of the TFR1 signaling pathway in UTMD-induced iron overload and ferroptosis, we next performed genetic loss- and gain-of-function experiments. *Tfr1/TFR1* short-hairpin RNA (*shTfr1/TFR1*) and *Tfr1/TFR1* overexpression (*Tfr1/TFR1-OE*) vectors were used to knock down or overexpress TFR1 in GBM cells, respectively. As expected, UTMD-induced increase of intracellular Fe^2+^ was reversed by TFR1 knockdown and enhanced by TFR1 overexpression in IR-treated GBM cells (Fig. 4c–e and Fig. S1c, d). Consistently, TFR1 knockdown abolished UTMD-caused generation of lipid ROS and total ROS, whereas TFR1 overexpression potentiated both (Fig. 4f–h and Fig. S1c, d). At the protein level, UTMD-induced upregulation of TFR1 and ACSL4 and downregulation of GPX4 were reversed by *shTfr1/TFR1* but further enhanced by *Tfr1/TFR1-OE* (Fig. 4i, j). Moreover, UTMD-caused MDA production and GSH depletion were abolished by TFR1 knockdown and aggravated by TFR1 overexpression (Fig. 4k, l). Functionally, TFR1 knockdown markedly attenuated UTMD-mediated inhibition of cell viability and induction of cell death in IR-treated GBM cells, whereas TFR1 overexpression markedly potentiated these effects (Fig. 4m, n and Fig. S1a, b). Together, these results indicated that TFR1 was involved in UTMD-induced intracellular Fe^2+^ accumulation and ferroptosis in IR-treated GBM cells.

### UTMD radiosensitized GBM via TFR1-dependent ferroptosis in mice

**Fig. 5:**
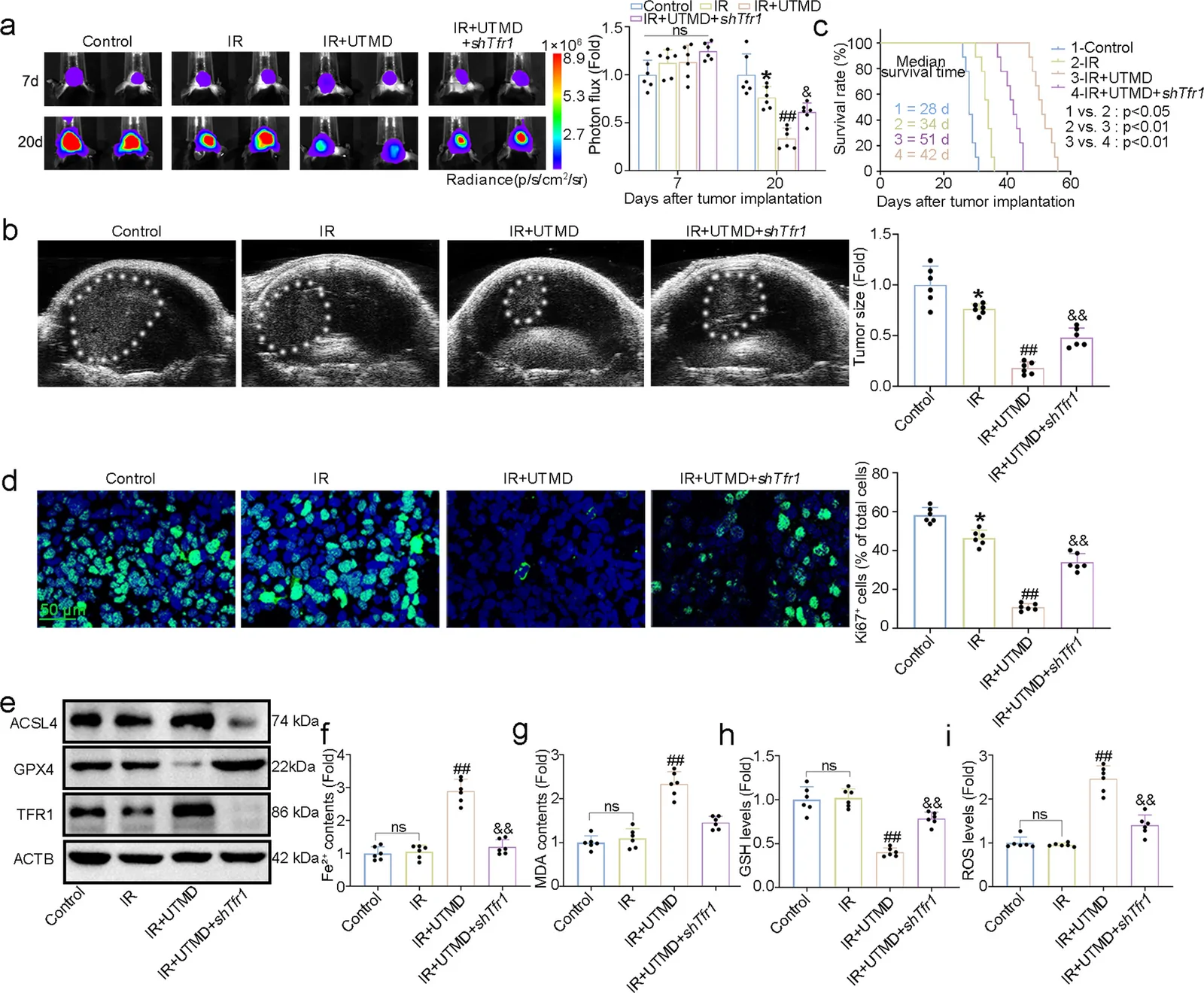
UTMD radiosensitized GBM via TFR1-dependent ferroptosis in C57BL/6J mice. Orthotopic GBM models were established in 6-week-old C57BL/6J mice by inoculating GL261 cells (1 × 10^5^ cells per mouse), with or without *shTfr1*-vector as described in the Materials and Methods. **a** In vivo bioluminescent imaging and quantification of intracranial GBM tumors. **b** Tumor size measured by US imaging; white circles demarcate the tumors. **c** Kaplan–Meier survival curves of GBM-bearing mice. Tumor tissues were collected on day 20 after implantation. **d** Immunofluorescence staining and the quantification of Ki67 (green) in intracranial GBM tumors. **e** The expression of ACSL4, GPX4, and TFR1 was detected by western blot. **f** Fe^2+^ levels were measured via a specific iron assay kit. Measurement of **g** MDA and **h** GSH levels by specific assay kits. **i** Detection of ROS levels using DCFH-DA staining followed by microplate reader. Data are expressed as the mean ± SD (n = 6); ^*^p < 0.05 vs. vehicle-treated control group; ^##^*p* < 0.01 vs. IR-treated group; ^&^*p* < 0.05, ^&&^*p* < 0.01 vs. IR and UTMD co-treated group; ns, not significant

**Fig. 6:**
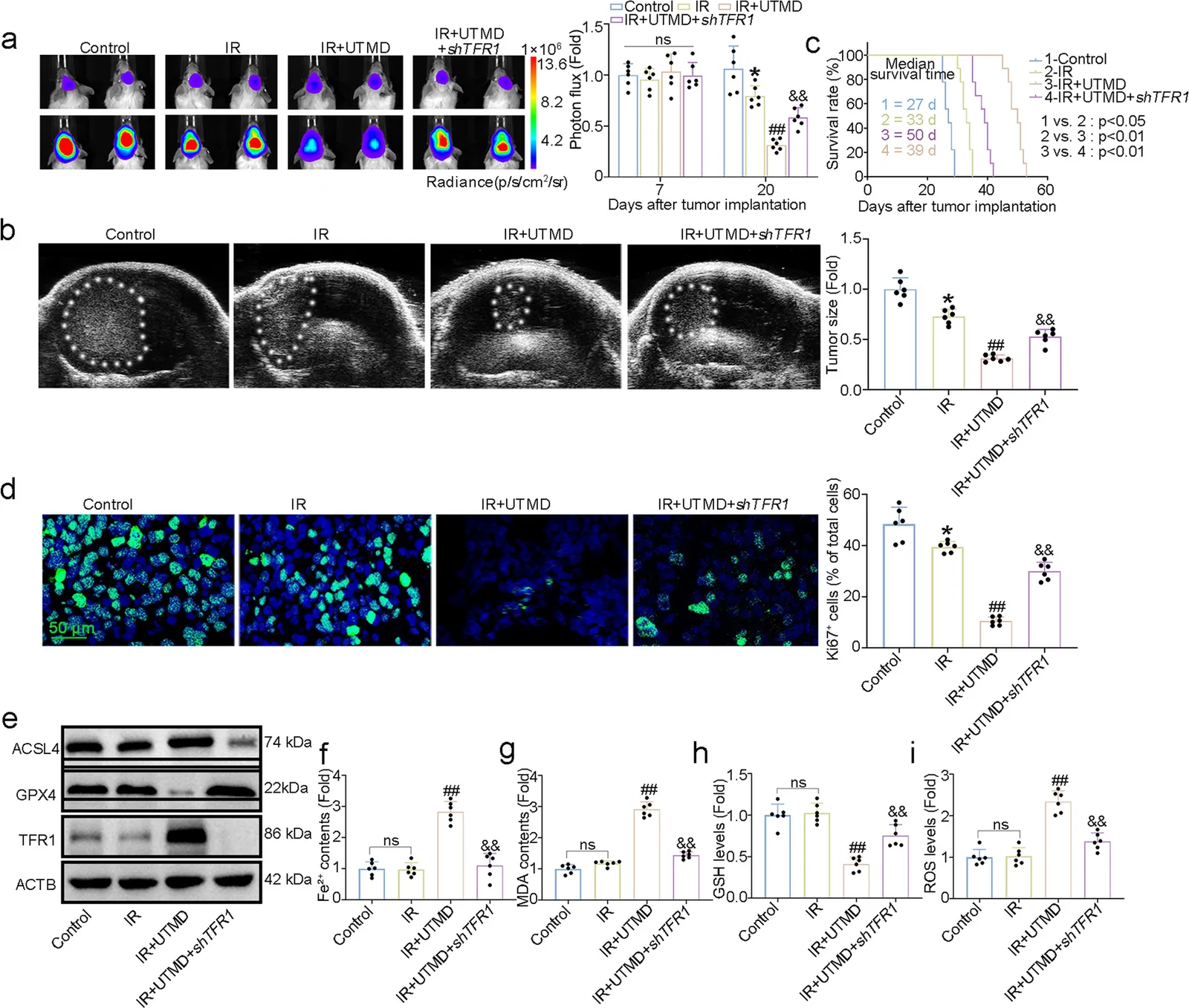
UTMD radiosensitized GBM via TFR1-dependent ferroptosis in NOD-SCID mice. Orthotopic GBM models were established in 6-week-old NOD-SCID mice by inoculating 1016B cells (2 × 10^4^ cells per mouse), with or without *shTFR1*-vector as described in the Materials and Methods. **a** In vivo bioluminescent imaging and quantification of intracranial GBM tumors. **b** Tumor size measured by US imaging; white circles demarcate the tumors. **c** Kaplan–Meier survival curves of GBM-bearing mice. **d** Immunofluorescence staining and quantification of Ki67 (green) in intracranial GBM tumors. Tumor tissues were collected on day 20 after implantation. **e** The expression of ACSL4, GPX4, and TFR1 was detected by western blot. **f** Fe^2+^ levels were measured via a specific iron assay kit. Measurement of **g** MDA and **h** GSH levels by specific assay kits. **i** Detection of ROS levels using DCFH-DA staining followed by microplate reader. Data are expressed as the mean ± SD (n = 6); ^*^p < 0.05 vs. vehicle-treated control group; ^##^*p* < 0.01 vs. IR-treated group; ^&&^*p* < 0.01 vs. IR and UTMD co-treated group; ns, not significant

To extend these mechanistic findings to a preclinical setting, we further explored the contribution of TFR1-mediated ferroptosis to UTMD-induced radiosensitization in vivo using two complementary mouse GBM models, namely GBM-bearing immunocompetent C57BL/6J mice and GBM-bearing immunodeficient NOD-SCID mice; the paired use of these models allowed us to verify that the observed mechanism operates regardless of the host immune status. The irradiation and UTMD regimen followed the schedule established in our earlier study [3]. In brief, tumor burden was evaluated by bioluminescence imaging on day 7 after implantation, after which mice were allocated to receive either vehicle control or daily IR (2 Gy) for 5 days, with or without UTMD administered every other day. Tumor progression and survival were monitored, and brain specimens were collected on day 20 for subsequent molecular analyses. Consistent with our prior work [3], UTMD potentiated the therapeutic efficacy of IR in GBM-bearing C57BL/6J mice: compared with IR alone, the combined UTMD and IR treatment substantially retarded intracranial tumor growth, as evidenced by reduced tumor size on US imaging and attenuated bioluminescence signals, decreased the proportion of Ki67-positive proliferating tumor cells, and significantly prolonged the survival of tumor-bearing mice (Fig. 5a–d). Moreover, the addition of UTMD to IR increased TFR1 and ACSL4 expression, intracellular Fe^2+^, MDA, and ROS contents, while decreasing GPX4 expression and GSH levels in IR-treated GBM-bearing C57BL/6J mice (Fig. 5e–i). These data indicated that UTMD triggered ferroptosis in vivo. Additionally, TFR1 knockdown abrogated UTMD-induced TFR1 expression and ferroptosis, thereby eliminating UTMD's radiosensitizing effect on GBM in C57BL/6J mice. Notably, the radiosensitizing effect of UTMD and the underlying TFR1-dependent ferroptotic mechanism were faithfully recapitulated in GBM-bearing NOD-SCID mice (Fig. 6a–i). Collectively, these results revealed that TFR1-mediated ferroptosis critically contributed to UTMD-induced radiosensitization of GBM in vivo.

## Discussion

Although radiotherapy remains a first-line therapy for GBM, its efficacy is severely limited by the intrinsic radioresistance of GBM cells, resulting in poor patient outcomes and necessitating the development of effective radiosensitizing strategies [24]. UTMD has emerged as a promising radiosensitizing strategy across diverse malignancies, including tumors of the prostate, bladder, liver, and breast [3, 25–29]. Compared to chemical radiosensitizers such as temozolomide, cisplatin, or targeted agents (e.g., poly(ADP-ribose) polymerase inhibitors, epidermal growth factor receptor inhibitors), UTMD offers several unique advantages: it is non-invasive, can be precisely targeted to tumor regions through US guidance, has minimal systemic toxicity, and can be repeated multiple times without cumulative side effects [30–32]. Moreover, unlike immunotherapies that require a functional immune system and may cause immune-related adverse events, UTMD can be applied in immunocompromised patients and does not depend on the host immune status [33]. The combination of UTMD with immunotherapy or targeted agents may further enhance therapeutic outcomes of IR through synergistic mechanisms, representing an exciting direction for future investigations [34]. Meanwhile, Peng et al. provided initial evidence that UTMD potentiates the cytotoxic effects of IR against GBM in a subcutaneous tumor xenograft model using nude mice [4]. Our recent work implied that UTMD potently augments radiosensitivity and extends survival duration in orthotopic GBM models [3]. These findings position UTMD as a promising adjuvant for GBM radiotherapy; however, limited data are available concerning the underlying mechanisms.

In the present study, for the first time, we found that UTMD strengthened the response of GBM cells to IR via inducing ferroptosis. Ferroptosis represents a newly identified form of programmed cell death orchestrated by ROS accumulation and iron-dependent lipid peroxidation, which is regarded as a pivotal determinant of glioma progression and treatment [8]. The cumulative published evidence supports the notion that ferroptosis induction is an effective approach to potentiate radiotherapy efficacy against GBM. Ye et al. found that ferroptosis induction via system xc − or GPX4 inhibition synergistically enhances IR efficacy in GBM by promoting cytoplasmic lipid peroxidation, without augmenting DNA damage or caspase activation [9]. Li et al. reported that protein arginine methyltransferase 1 depletion sensitizes glioma stem cell-like cells to radiotherapy, an effect that is mitigated by Fer-1 and accompanied by inhibition of ferroptosis [11]. Zhu et al. showed that downregulation of regulator of G-protein signaling 4 induces ferroptosis, subsequently promoting glioma cell sensitivity to radiotherapy and temozolomide [10]. Moreover, several studies have shown that US increases total and lipid ROS levels, and decreases GSH contents in breast cancer cells, while ferroptosis inhibitors (Fer-1 or DFO) significantly alleviate the cytotoxicity induced by US [12]. In pancreatic cancer cells, US decreases GPX4 expression, thereby potentiating oridonin-induced inhibition of tumor growth by inducing ferroptosis [13]. These findings implicate US-mediated ferroptosis induction as a novel therapeutic strategy for oncologic intervention. Our findings extended this body of evidence by showing that UTMD, a physical therapeutic modality, can trigger ferroptosis to enhance radiosensitivity in GBM, thereby providing a novel mechanistic link between ultrasound-based physical intervention and iron-dependent cell death.

Furthermore, our findings implicated TFR1 as a key regulator of UTMD-driven ferroptosis in IR-treated GBM cells. TFR1 is a type II transmembrane glycoprotein localized at the cell membrane that governs cellular iron homeostasis by mediating iron uptake. TFR1 is highly expressed in many malignancies including GBM and represents a promising therapeutic target [35]. Recently, TFR1 has been identified as a key regulator of ferroptosis by controlling the intracellular iron contents [15]. Studies have reported that coxsackievirus B3 or pseudorabies virus increases TFR1 expression, causing substantial release of free iron and thereby promoting lipid peroxidation and ferroptosis; suppression of ferroptosis with Fer-1, DFO or genetic interference prevents cell death and attenuates viral replication [23, 36]. More recently, Lu et al. confirmed that brucine induces ferroptosis by promoting iron importation through increasing TFR1 expression, not via inhibition of ferritin or FPN [19]. We likewise observed that UTMD markedly increased the intracellular total iron and Fe^2+^ levels in a TFR1-dependent manner. Meanwhile, TFR1 downregulation abolished UTMD-induced intracellular iron overload, thereby attenuating UTMD-triggered ferroptosis and radiosensitization in GBM, which were augmented by TFR1 overexpression. The present study therefore indicated that TFR1 was a critical mechanistic link between UTMD and ferroptosis induction. A particularly noteworthy aspect of our findings is the mechanistic insight into how UTMD regulates TFR1. Previous studies have supported the notion that the mechanical forces generated by UTMD, including acoustic cavitation, microstreaming, and shear stress, may directly modulate cell-surface ion channels/receptors expression or endocytosis at the cell membrane [37–40]. This hypothesis is supported by our observation that UTMD alone upregulated TFR1 expression without inducing ferroptosis, suggesting that TFR1 upregulation was an early event that primed cells for ferroptosis upon subsequent IR exposure. In addition, several lines of evidence point to the involvement of canonical mechanotransduction signaling pathways in UTMD-induced regulation of cell-surface ion channels/receptors. Microbubble cavitation generates acoustic radiation forces, microstreaming, and transient membrane deformation, which cells convert into biochemical signals through mechanosensitive machinery, including integrin ligation, mechanosensitive ion channels, and intracellular Ca^2^⁺ transients [41, 42]. Whether and how the mechanotransduction signaling pathways contribute to UTMD-mediated TFR1 upregulation warrants further investigation.

Looking ahead, artificial intelligence (AI) technologies are expected to play a pivotal role in deciphering the precise mechanisms through which UTMD regulates TFR1, as well as in accelerating the development of targeted drugs. AlphaFold, the AI protein structure prediction system developed by DeepMind, has demonstrated remarkable accuracy in predicting protein structures, and its extension, AlphaFold 3, has been increasingly applied to model protein–protein interactions and identify potential drug targets [43–45]. The application of AlphaFold to analyze TFR1 and its known interacting partners (e.g., transferrin and HFE), as well as functionally connected ferroptosis regulators, could provide valuable structural insights into the ferroptotic machinery and facilitate the rational design of small-molecule modulators targeting TFR1-mediated iron metabolism. Furthermore, AI-driven molecular dynamics simulations could help elucidate, at the atomic level, how UTMD-induced mechanical forces might modulate TFR1 conformation and function, and could guide the rational design of TFR1-targeted therapeutics. Integration of these computational approaches with our experimental findings could significantly accelerate the development of optimized UTMD protocols and TFR1-targeted combination therapies for GBM.

From a translational perspective, UTMD offers significant advantages for GBM treatment; however, there are still some challenges to its practical application (e.g., cranial penetration and targeting of MBs). Because bone tissue strongly attenuates the propagation of US, transcranial US imaging is currently performed through ‘acoustic windows’, regions of the skull where the bone is thin enough to permit adequate signal transmission [46]. As radiotherapy is almost invariably delivered after surgical resection in GBM patients, the presence of a postoperative cranial window or bone defect would substantially reduce the barrier effect of the skull on UTMD delivery [47]. Meanwhile, recent advances in US technology have further shown that skull attenuation can be effectively overcome using phased-array transducers operating at approximately 650 kHz, enabling precise targeting of centrally located deep brain tumors [48, 49]. Moreover, clinically approved US contrast agents (e.g., SonoVue) have been shown to reach intracranial tumors after intravenous administration, providing a feasible pathway for MB delivery [50, 51]. Collectively, these considerations indicate that the combination of UTMD with fractionated radiotherapy, the current standard of care for GBM, represents a highly translatable therapeutic strategy.

This study has several limitations. First, while we demonstrated that UTMD upregulated TFR1 expression, the precise molecular mechanism linking UTMD-mediated mechanical forces to TFR1 transcriptional upregulation remains to be fully elucidated. Second, although we employed two distinct GBM cell lines and two orthotopic mouse models, additional validation in patient-derived xenograft models or genetically engineered mouse models would further strengthen the translational relevance of our findings. Third, the long-term safety and optimal dosing schedule of UTMD for GBM treatment in clinical settings require further investigation. Finally, the potential interactions between UTMD and the tumor immune microenvironment, which may influence therapeutic outcomes, were not addressed in the present study.

**Fig. 7:**
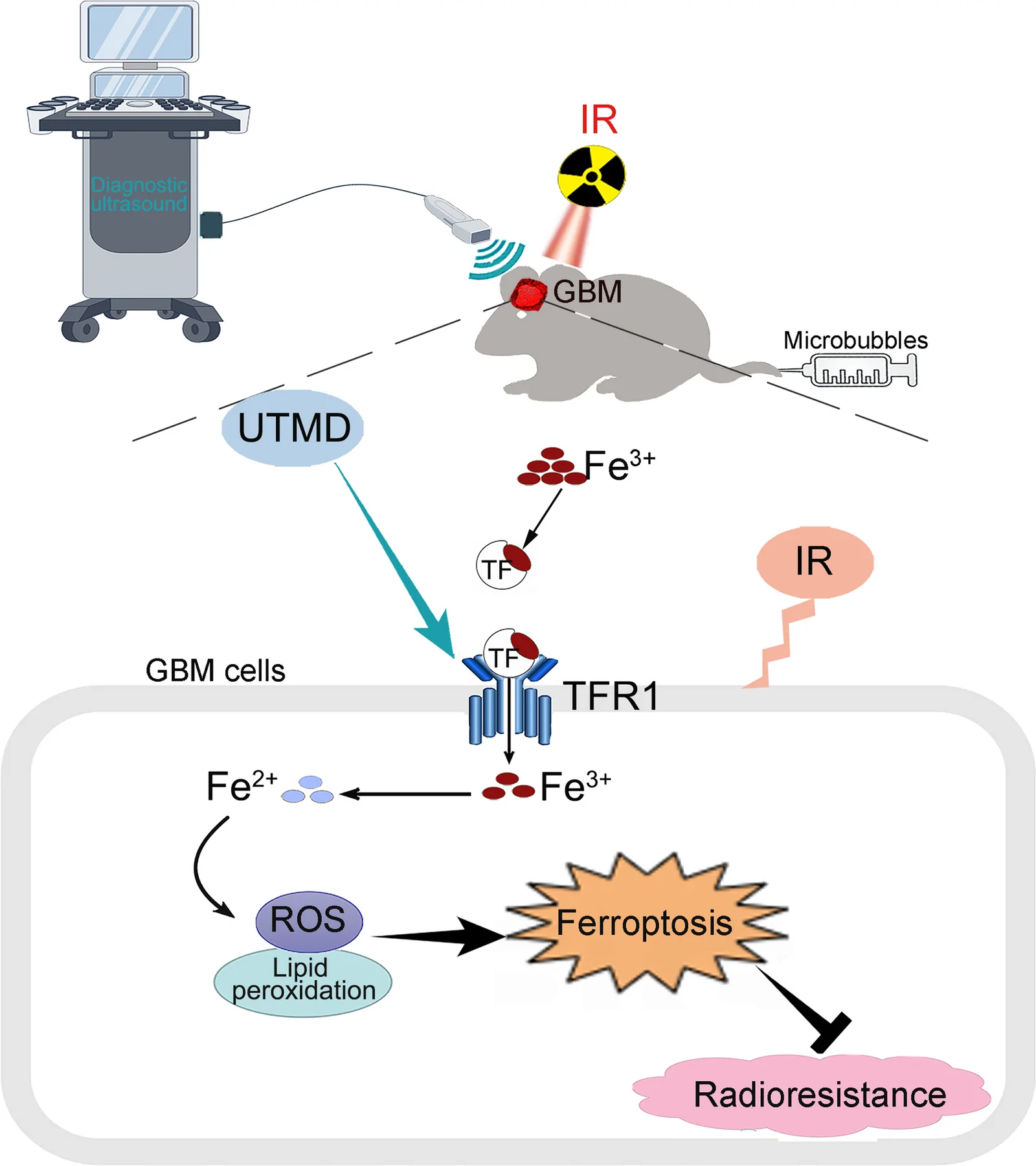
UTMD enhanced the radiosensitivity of GBM by inducing TFR1-mediated ferroptosis. UTMD sensitizes GBM cells to IR via the proposed signaling pathways: UTMD upregulates TFR1 expression, thereby increasing intracellular iron/Fe^2+^, leading to lipid peroxidation, subsequently triggering ferroptosis and ultimately enhancing the radiosensitivity of GBM; TF: transferrin

In summary, this work revealed that UTMD sensitized GBM to radiotherapy, at least partially, by triggering TFR1-dependent ferroptosis (Fig. 7), thereby delineating a novel mechanism underlying its radiosensitizing action. Our findings extend previous observations and fill an important mechanistic gap in the field. These data not only highlight UTMD as a potential therapeutic adjuvant for GBM radiotherapy but also provide a biological foundation for its broader clinical application.

## Materials and methods

### Preparation of MBs

The MB formulation (designated ZHIFUXIAN) was generated in-house according to our previously established protocol [52]. In brief, a lipid mixture composed of dipalmitoyl phosphatidylglycerol, distearoyl phosphatidylcholine, and polyethylene glycol-4000, combined at a mass ratio of 30:30:3000, was dissolved in ultrapure water at 60 °C until a clear solution was obtained. Thereafter, 1 mL of the suspension was dispensed into each 2 mL vial and lyophilized. The lyophilized cake in each vial was rehydrated with 1 mL of a medium containing glycerin, propylene glycol, and 50% glucose (volume ratio 1:1:8), and the vial headspace was flushed with perfluoropropane gas. After airtight sealing, the vials were agitated with a shaker (AT & M, Beijing, China) at 4,000–5,500 cycles per minute for 60 s. The resulting ZHIFUXIAN MBs had a concentration of approximately 4–9 × 10⁹ MBs/mL and a mean diameter of 2.13 μm.

### Cell treatments

GL261 and 1016B cells in the logarithmic growth phase received UTMD (1.2 W/cm^2^ ultrasonic intensity, 200 μL/mL MBs, 10% duty cycle, 60 s) and were then irradiated with 2 Gy IR delivered by an RS-2000 irradiator (Rad Source Technologies, Atlanta, GA; 160 kVp X-rays, 0.3-mm copper filter, 1 Gy/min dose rate) as reported before [3]. Where indicated, cells were additionally incubated with Fer-1 (50 μM), erastin (10 μM), RSL3 (2.5 μM), Z-VAD-FMK (50 μM), 3-MA (5 mM), Nec-1 (20 μM), DFO (500 μM), FAC (500 μM), VIT-2763 (2 μM), or Fur (10 μM) for 1 h before exposure to IR alone or IR combined with UTMD, and were harvested 24 h later.

### Animal treatments

Orthotopic GBM models were established as per our previous description by inoculating GL261 or 1016B cells into the right caudate putamen of 6-week-old C57BL/6J or NOD-SCID mice [53]. In brief, luciferase-expressing GL261 or 1016B cells were first transfected with lentiviral vectors carrying *shTfr1/TFR1*. C57BL/6J and NOD-SCID mice were then stereotactically injected with 1 × 10^5^ GL261 cells or 2 × 10^4^ 1016B cells per mouse, respectively, into the right hemisphere of the brain (coordinates: 1 mm anterior, 2.5 mm lateral to bregma, 3.5 mm depth). 7 days after implantation, the mice were anesthetized via intraperitoneal injection of 1% pentobarbital sodium (50 mg/kg), followed by continuous intravenous infusion of MBs (300 μL per mouse). UTMD was performed with a VINNO 70 scanner (Vinno Technology Co. Ltd, Suzhou, China) fitted with an X4-12L linear array transducer, which was positioned over the glioma with US transmission gel. The US parameters were: 3 MHz frequency, 50 Hz pulse repetition frequency, 26-cycle pulse length, MI ≈ 0.8, pulse/interval time of 0.62/2 s, and 1 cm focal depth, applied for 5 min. Immediately after UTMD, whole-brain irradiation (2 Gy) was delivered with the RS-2000 irradiator (Rad Source Technologies, Atlanta, GA). This combined regimen (UTMD every other day plus daily IR) was given for 5 days, reaching a total IR dose of 10 Gy. In previous studies on orthotopic GBM-bearing mice, total IR doses of 2–10 Gy have been reported, with 10 Gy delivered in 5 fractions (2 Gy/fraction) being the most widely used schedule [54–56]; this schedule was therefore selected for our in vivo experiments. Furthermore, the regimen of UTMD every other day was adopted in line with our previous reports [3] based on the following considerations: (1) UTMD requires intravenous administration of MBs followed by ultrasound exposure, which necessitates longer preparation and execution time compared with IR delivery, and daily UTMD would be logistically challenging and could increase ultrasound-related side effects; (2) this interval allows a washout period between UTMD sessions, reducing potential cumulative acoustic toxicity while maintaining the radiosensitizing effect; and (3) no significant toxicity, such as weight loss, neurological symptoms, or tissue damage, was observed with this regimen, supporting its safety and feasibility. Tumor growth was monitored by the IVIS bioluminescence imaging system (PerkinElmer, Waltham, MA, USA), and quantification was carried out using Living Image Software (PerkinElmer, Waltham, MA, USA). Animals were sacrificed on day 20 post-transplantation or upon neurological symptom manifestation. Brains were collected and snap-frozen in liquid nitrogen and stored at −80 °C until processing.

### Statistical analyses

All quantitative data are expressed as the mean ± standard deviation (SD) from three independent experiments. Comparisons were performed using the t-test or one-way analysis of variance in SPSS 22.0 (SPSS Inc., Chicago, IL, USA). Survival was evaluated by Kaplan–Meier analysis with the log-rank test. A p value < 0.05 was considered statistically significant.

Full descriptions of additional materials and methods, including detailed information on the antibodies, reagents, and instruments used in this study, are given in the Supporting Information.

## Supplementary Information

Supplementary Material 1.

## Data Availability

All data generated or analyzed during this study are included in this article and its supplementary information files. Further datasets and materials are available from the corresponding authors upon reasonable request.

## Abbreviations

3-MA: 3-Methyladenine
ACSL4: Acyl-CoA synthetase long-chain family member 4
ACTB: β-Actin
AI: Artificial intelligence
CCK-8: Cell counting kit-8
C11-BODIPY: BODIPY™ 581/591 C11
DAPI: 4',6-Diamidino-2-phenylindole
DFO: Deferoxamine
DMEM: Dulbecco’s modified Eagle’s medium
FAC: Ferric ammonium citrate
FBS: Fetal bovine serum
Fer-1: Ferrostatin-1
FTH: Ferritin heavy chain
FTL: Ferritin light chain
FPN1: Ferroportin 1
Fur: Fursultiamine
GBM: Glioblastoma multiforme
GPX4: Glutathione peroxidase 4
GSH: Glutathione
IR: Ionizing radiation
MBs: Microbubbles
MDA: Malondialdehyde
MFI: Mean fluorescence intensity
Nec-1: Necrostatin-1
oxC11-BODIPY: Oxidized C11-BODIPY
PBS: Phosphate-buffered saline
PI: Propidium iodide
ROS: Reactive oxygen species
RSL3: RAS-selective lethal 3
TFR1: Transferrin receptor 1
*Tfr1/TFR1-OE*: *Tfr1/TFR1* Overexpression
*shTfr1/TFR1*: *Tfr1/TFR1* Short-hairpin RNA
US: Ultrasound
UTMD: Ultrasound-triggered microbubble destruction
Z-VAD-FMK: Z-Val-Ala-Asp(OMe)-fluoromethylketone
