# Supplementary material for: Induction of transferrin receptor 1-mediated ferroptosis by ultrasound-triggered microbubble destruction sensitizes glioblastoma to radiotherapy
Source: Mol Biomed. 2026 Sep 18;7:178. doi: 10.1186/s43556-026-00572-8 (PMC13589017; doi:10.1186/s43556-026-00572-8)
Supplement: Supplementary file 1 [file 43556_2026_572_MOESM1_ESM.docx]

**Supplementary Materials for:**

**Induction of transferrin receptor 1-mediated ferroptosis by ultrasound-triggered microbubble destruction sensitizes glioblastoma to radiotherapy**

Ying He^1,2†^, Xunhu Dong^4,5†^, Xiaolu Lu^6†^, Qiong Zhu^2†^, Qingqing Miao^1^, Zexuan Yan^1^, Qingning Zhao^1^, Hongying Chen^4^, Min Qin^4^, Yuehua Yi^6^, Jiaqi Li^1^, Dan Yang^7^, Hanyun Zhang^8^, Yali Xu^2*^, Mingliang Chen^3,4*^, Xiuwu Bian^1,6*^, Xing Hua^2*^

†These authors contribute equally to this work

^1^Institute of Pathology and Southwest Cancer Centre, Southwest Hospital, Army Medical University (Third Military Medical University) and the Key Laboratory of Tumor Immunopathology, The Ministry of Education of China, Chongqing, 400038, China.

^2^Department of Ultrasound, Xinqiao Hospital, Army Medical University (Third Military Medical University), Chongqing, 400037, China.

^3^Department of Clinical Nutrition, Southwest Hospital, Army Medical University (Third Military Medical University), Chongqing, 400038, China.

^4^Institute of Toxicology, School of Military Preventive Medicine, Army Medical University (Third Military Medical University), Chongqing, 400038, China.

^5^Institute of Burn Research, State Key Laboratory of Trauma, Burn and Combined Injury, Southwest Hospital, Third Military Medical University (Army Medical University), Chongqing 400038, China.

^6^School of Basic Medical Sciences, Southern Medical University, Guangzhou, 510151, China.

^7^Department of Ultrasound of Jiangbei Campus, Southwest Hospital, Army Medical University (Third Military Medical University), Chongqing, 400020, China.

^8^School of Basic Medical Sciences, Army Medical University (Third Military Medical University), Chongqing 400038, China

*Correspondence: Yali Xu, E-mail: xuyal1976@tmmu.edu.cn; Mingliang Chen, E-mail: chenml@tmmu.edu.cn; Xiuwu Bian, E-mail: bianxiuwu@tmmu.edu.cn;

Xing Hua, E-mail: huaxing_ultra@tmmu.edu.cn.

**1. Materials and Methods**

**1.1 Antibodies and reagents**

Dulbecco’s modified Eagle’s medium (DMEM, 11965167) and fetal bovine serum (FBS, [26010074](https://www.thermofisher.com/order/catalog/product/26010074)) were purchased from Gibco Invitrogen Corp (USA). CCK-8 kit (CK04) was purchased from Dojindo Laboratories (Kumamoto, Japan). PI (ST512), phosphate-buffered saline (PBS, C0221A), 4',6-diamidino-2-phenylindole (DAPI, C1005), RIPA (P0013B), bradford protein assay kit (P0006), MDA assay kit (S0131M), GSH assay kit (S0053), DCFH-DA (S0033) were obtained from the Beyotime Institute of Biotechnology (Shanghai, China). C11-BODIPY (D3861) was acquired from Invitrogen (Carlsbad, CA, USA). FerroOrange (36104) was from Cell Signaling Technology (Beverly, MA, USA). Dimethyl sulfoxide (DMSO, D2650) and iron assay kit (MAK025) were purchased from Sigma-Aldrich (St. Louis, MO, USA). Erastin (HY-15763), Fer-1 (HY-100579), RSL3 (HY-100218A), Z-VAD-FMK (HY-16658B), 3-MA (HY-19312), Nec-1 (HY-15760), VIT-2763 (HY-112220), Fur (HY-B2082), DFO (HY-B1625), and FAC (HY-B1645) were purchased from Med Chem Express (Monmouth Junction, NJ, USA).

The details of all antibodies used in this study, including host species, clonality, suppliers, catalog numbers, working dilutions, and applications, are summarized in **Table S1**.

**Table S1. Antibodies used in this study**

| **Antibody** | **Host species** | **Clonality** | **Dilution** | **Supplier** | **Catalog No.** |
| --- | --- | --- | --- | --- | --- |
| Anti-TFR1 | Rabbit | Polyclonal | 1:2,000  (WB) | Proteintech  (Wuhan, China) | 32333-1-AP |
| Anti-FPN1 | Rabbit | Polyclonal | 1:1,000  (WB) | Proteintech  (Wuhan, China) | 26601-1-AP |
| Anti-ACSL4 | Rabbit | Polyclonal | 1:5,000  (WB) | Proteintech  (Wuhan, China) | 22401-1-AP |
| Anti-FTH | Mouse | Monoclonal | 1:1,000  (WB) | Proteintech  (Wuhan, China) | 60875-5-Ig |
| Anti-FTL | Rabbit | Polyclonal | 1:2,000  (WB) | Proteintech  (Wuhan, China) | 10727-1-AP |
| Anti-GPX4 | Mouse | Monoclonal | 1:1,000  (WB) | Proteintech  (Wuhan, China) | 67763-1-Ig |
| Anti-β-actin (ACTB) | Mouse | Monoclonal | 1:1,000  (WB) | Beyotime  (Shanghai, China) | AF2811 |
| Anti-Ki67 | Rabbit | Polyclonal | 1:100  (IF) | Abcam  (Cambridge, UK) | ab15580 |
| HRP-conjugated goat anti-rabbit IgG | Goat | Polyclonal | 1:5,000  (WB) | Thermo Scientific (Waltham, MA, USA) | 31455 |
| HRP-conjugated goat anti-mouse IgG | Goat | Polyclonal | 1:5,000  (WB) | Thermo Scientific (Waltham, MA, USA) | 31340 |
| Alexa Fluor 488 goat anti-mouse IgG (H+L) | Goat | Polyclonal | 1:500  (IF) | Invitrogen  (Carlsbad, CA, USA) | A11029 |

WB, western blotting; IF, immunofluorescence.

**1.2 Cell culture**

Murine GL261 GBM cells (FH1097) were purchased from Fuheng Biology (Shanghai, China) and 1016B human GBM cells were generated from two surgical specimens of GBM patients (Southwest Hospital, Army Medical University, China) in our laboratory[1]. Cells were cultured in DMEM supplemented with 10% FBS at 37°C in a humidiﬁed atmosphere containing 5% CO_2_. All experiments were performed following 3–6 passages when the cells reached ~80–90% confluence.

**1.3 Animals**

6-week-old C57BL/6J and NOD-SCID mice were purchased from Hunan SJA Laboratory Animal Co. Ltd. (Hunan, China) and housed at 22 ± 2℃ under a 12 h light/dark cycle with *ad libitum* access to food and water.

**1.4 Cell viability measurement**

The CCK-8 kit was employed to evaluate cell viability per established protocols[2]. Briefly, GL261 or 1016B cells were seeded in a 96-well microplate (Corning Life Sciences; 3650) at a density of 5,000 cells/well and then subjected to IR at 2 Gy combined with or without UTMD, respectively. After 24 h, CCK-8 solution was added (20 μL/well) and the plate was incubated at 37°C for 1.5–2.0 h. Absorbance at 450 nm (Safire II microplate reader; Tecan Group Ltd., Männedorf, Switzerland) was used to assess the cell viability. The percent cell viability was normalized to the control group's 450 nm absorbance (set as 100%).

**1.5 Cell death assay**

Cell death was measured by PI staining as previously reported[3]. Briefly, cells were trypsinized with 0.25% trypsin for 1 min, harvested and resuspended in PBS. After incubation with PI staining solution (0.5 mL, 10 mg/mL) at 37℃ for 30 min in darkness, cell death was assessed by flow cytometry (BD FAC Scan Flow cytometer).

**1.6 Measurement of intracellular Fe^2+^ and total iron levels**

The intracellular Fe^2+^ and total iron levels were assessed by an iron assay kit per manufacturer's instructions. Specifically, GBM cells or tissues were lysed in 4–10 volumes of iron assay buffer, followed by centrifugation at 16,000 × g (10 min, 4°C) to remove insoluble components. Subsequently, the samples were diluted to 100 μL with assay buffer in 96-well plates and incubated with either 5 μL iron assay buffer (for Fe^2+^) or iron reducer (for total iron) for 30 min at 25°C. Finally, Fe^2+^ and total iron concentrations were measured at 593 nm using an Infinite™ M200 Microplate Reader (Tecan Group Ltd.)..

Meanwhile, FerroOrange was also applied to detect the intracellular Fe^2+^ levels. After the indicated treatments, GBM cells were incubated with 1 µM FerroOrange at 37℃ for 15 min, then washed and stained with or without DAPI for 5 min. Finally, cells were washed with PBS and Fe^2+^ contents were quantified by ZEISS LSM900 confocal laser scanning microscope (ZEISS, Germany) and flow cytometry (BD FAC Scan Flow cytometer), following the supplier’s protocol.

**1.7 Lipid peroxidation measurement**

C11 BODIPY was used to determine the production of lipid peroxidation. At the end of the indicated treatments, GBM cells were washed with PBS and stained with C11 BODIPY (2 μM) at 37℃ for 30 min. Thereafter, cells were washed by PBS and incubated with or without DAPI for 5 min. Finally, the levels of lipid peroxidation were quantified by ZEISS LSM900 confocal laser scanning microscope (ZEISS, Germany) and flow cytometry (BD FAC Scan Flow cytometer), following the supplier’s protocol.

**1.8 ROS assessments**

ROS contents were measured using DCFH-DA per the manufacturer's instructions as previously reported[4]. GBM cells or tissues were collected and incubated with DCFH-DA (10 μM) at 37°C for 30 min under dark conditions, followed by three gentle rinses with warm PBS. The fluorescence intensity corresponding to total ROS was then detected by flow cytometry (BD FAC Scan Flow cytometer) or Infinite™ M200 Microplate Reader (Tecan Group Ltd.)

**1.9 Measurement of GSH and MDA contents**

At the end of treatment, GBM cells/tissues were collected and lysed with RIPA lysis buffer. The levels of GSH and MDA were detected by commercial assay kits according to the manufacturer's protocols.

**1.10 Western blot analysis**

Cells and brain tumors were collected, lysed and subjected to western blotting as described previously[5]. Briefly, 40-150 µg of protein was resolved by 10-15% SDS-PAGE and then electroblotted onto polyvinylidene difluoride membranes for western blot analysis. Blots were probed with 1:1,000-1:5,000-diluted primary antibodies overnight at 4°C, followed by HRP-conjugated secondary antibodies (1:5000). The protein bands were visualized using an enhanced chemiluminescence system, and densitometric analysis was performed using Scion Image-Release Beta 4.02 software ([http://scion-corporation.software.informer.com](http://scion-corporation.software.informer.com/)).

**1.11 Transmission electron microscopy**

GBM cells were fixed in 2% paraformaldehyde and 0.1% glutaraldehyde in 0.1 M sodium cacodylate for 2 h, post-fixed with 1% OsO4 for 1.5 h. After washing, cells were stained with 3% aqueous uranyl acetate for 1 h. Subsequently, the samples were washed again, dehydrated with graded alcohol, and embedded in Epon-Araldite resin (Canemco & Marivac, 034). Ultrathin sections were cut by an ultramicrotome (Reichert-Jung, Inc., Cambridge, UK), counterstained with 0.3% lead citrate, and examined on a transmission electron microscope (model no.: EM420; Koninklijke Philips Electronics N.V., Amsterdam, The Netherlands).

**1.12 Lentiviral construction and cell transfection**

The lentiviral vectors containing *shTfr1/TFR1* and *Tfr1*/*TFR1* were purchased from Gene Chem (Shanghai, China). The processes of transduction and the establishment of stable TFR1 knockdown or overexpression cell lines were executed according to the manufacturer's instructions.

**1.13** **Immunofluorescence analysis**

For immunofluorescence analysis, brain tumors were harvested and sectioned at a thickness of 6-8 μm. The sections were washed by PBS, permeabilized with 0.3% (v/v) Triton X-100 for 10 min and blocked in 3% BSA for 1 h at room temperature. After that, sections were incubated with anti-Ki67 antibody (1:100) overnight at 4°C. Then, the sections were washed and incubated for 2 h at room temperature with Alexa Fluor® 488 goat anti-mouse IgG (H+L) antibody (1:500). Nuclei were counterstained with DAPI for 10 min at room temperature. Finally, the sections were mounted on glass slides. All digital images were acquired using a ZEISS LSM900 confocal laser scanning microscope (ZEISS, Germany) under uniform settings (laser intensity, pinhole, gain, offset) to ensure comparability across groups.

**1.14 Ultrasound detection of orthotopic GBM**

The orthotopic GBM was imaged using a Vevo 2100 high-frequency ultrasound system (VisualSonics, Toronto, Canada) with an MS-400 linear array transducer (30 MHz center frequency) per our prior description^[5]^. In brief, GBM-bearing mice were prepared for imaging by intraperitoneal anesthesia with 1% pentobarbital sodium (50 mg/kg), followed by shaving the imaging site and application of warm ultrasound gel to ensure optimal image acquisition. A 13×12 mm field of view (width×depth) was used. Imaging was performed in the prone position with the transducer in the coronal plane (from vertex to caudal) and sagittal plane (from midline to lateral), both in a slow, controlled manner. Optimization of settings yielded 50-μm resolution with frame rates in 2D up to 628 frames per second for a 4 × 4 mm field of view per manufacturer recommendations. Variability was minimized by maintaining constant imaging parameters, using an experienced operator for all examinations, and performing triplicate measurements at each site.

**Figure S1. Flow cytometry analyses of cell death, ROS, Fe^2+^ and lipid ROS related to Fig. 1-4.** Cells were subjected to UTMD (1.2 W/cm^2^, 200 μL/mL MBs, 10% duty cycle, 60 s) and then irradiated with 2 Gy. Where indicated, cells were pre-incubated for 1 h with Fer-1 (50 μM), erastin (10 μM), RSL3 (2.5 μM), DFO (500 μM), or FAC (500 μM) before IR, UTMD, or combined treatment for another 24 h. TFR1 was knocked down or overexpressed using *shTfr1/TFR1* or *Tfr1/TFR1-OE* lentiviruses as described in the Materials and Methods, after which cells were treated with IR or IR plus UTMD for a further 24 h. Cell death of **(a)** GL261 and **(b)** 1016B cells was determined with PI staining by ﬂow cytometry as described in the Materials and Methods. Total ROS, lipid ROS and Fe^2+^ levels in **(c)** GL261 and **(d)** 1016B cells were determined with DCFH-DA, C11-BODIPY, or FerroOrange staining by ﬂow cytometry as described in the Materials and Methods.

**References:**

[1]	Cao M, Cai J, Yuan Y, et al. A four-gene signature-derived risk score for glioblastoma: prospects for prognostic and response predictive analyses. Cancer Biol Med. 2019; 16(3): 595-605. https://doi.org/10.20892/j.issn.2095-3941.2018.0277.

[2]	Chen ML, Zhu XH, Ran L, Lang HD, Yi L, Mi MT. Trimethylamine-N-Oxide Induces Vascular Inflammation by Activating the NLRP3 Inflammasome Through the SIRT3-SOD2-mtROS Signaling Pathway. J Am Heart Assoc. 2017; 6(9): e006347. https://doi.org/10.1161/jaha.117.006347.

[3]	Chen ML, Dong XH, Deng H, et al. Targeting TRPV1-mediated autophagy attenuates nitrogen mustard-induced dermal toxicity. Signal Transduct Target Ther. 2021; 6(1): 29. https://doi.org/10.1038/s41392-020-00389-z.

[4]	Dong XH, He Y, Hu XF, et al. Targeting LINC00707 by vitamin D3 attenuates nitrogen mustard-caused dermal toxicity through inhibiting ferroptosis. Redox Biol. 2025; 83: 103628. https://doi.org/10.1016/j.redox.2025.103628.

[5]	He Y, Dong XH, Zhu Q, Xu YL, Chen ML, Liu Z. Ultrasound-triggered microbubble destruction enhances the radiosensitivity of glioblastoma by inhibiting PGRMC1-mediated autophagy in vitro and in vivo. Mil Med Res. 2022; 9(1): 9. https://doi.org/10.1186/s40779-022-00369-0.
